# Endovascular treatment of thoracoabdominal aortic aneurysms with custom-made endografts

**DOI:** 10.1590/1677-5449.202401752

**Published:** 2025-11-17

**Authors:** André Rodrigo Miquelin, Daniel Gustavo Miquelin, Fernando Reis, Luiz Fernando Reis, José Maria Pereira de Godoy

**Affiliations:** 1 Faculdade de Medicina de São José do Rio Preto – FAMERP, Departamento de Cirurgia Vascular e Endovascular, São José do Rio Preto, SP, Brasil.

**Keywords:** endovascular treatment, thoracoabdominal aneurysm, branched endografts, fenestrated endografts, visceral branches, tratamento endovascular, aneurisma toracoabdominal, endopróteses ramificadas, endopróteses fenestradas, ramos viscerais

## Abstract

Endovascular treatment of thoracoabdominal aortic aneurysms (TAAAs) is a less invasive alternative, with reduced mortality and morbidity compared to open repair. However, reports on the results of this procedure in Brazil are scarce. This article reports the first institutional experience with endovascular treatment of TAAAs using branched and fenestrated endografts. Four patients with TAAA were included in this report. The devices used were custom-made by Braile, tailored to each patient’s anatomy based on computed tomography (CT) imaging. A total of 10 visceral branches were required to maintain the patency of the visceral arteries. CT follow-up was performed 2 months after the procedure. All implants were successfully completed. The fenestration/branch interfaces were secured with covered, balloon-expandable, and self-expanding peripheral stents in 8 of the 10 target branches. The remaining 2 branches were preserved with openings and free flow. Post-procedural angiography demonstrated correct positioning of the endografts and stents in the visceral arteries, with patency maintained in 9 of the 10 target branches. Among the complications, one selective catheterization of the celiac trunk was not possible at the first attempt. However, there was no mesenteric ischemia, and a peripheral stent was successfully implanted in the celiac trunk in a second procedure. All patients had a complication-free recovery. Follow-up CT demonstrated complete aneurysm exclusion with no leaks and visceral perfusion was maintained in 7 of the 8 target branches. It was concluded that endovascular treatment of TAAA with custom-made endografts is feasible and may become a treatment of choice for patients with challenging anatomical lesions.

## Resumo

O tratamento endovascular de aneurismas da aorta toracoabdominal (AATAs) é uma alternativa menos invasiva, com menor mortalidade e morbidade em comparação ao reparo aberto. No entanto, são escassos os relatos sobre os resultados desse procedimento no Brasil. Este artigo relata a primeira experiência institucional com o tratamento endovascular de AATAs utilizando endopróteses ramificadas e fenestradas. Quatro pacientes com AATA foram incluídos neste relato. As endopróteses foram confeccionadas sob medida pela Braile, de acordo com as características anatômicas de cada paciente, avaliadas por tomografia computadorizada (TC). Foram necessários 10 ramos viscerais para manter a perviedade das artérias viscerais. O acompanhamento por TC foi realizado 2 meses após o procedimento. Todos os implantes foram bem-sucedidos. As interfaces de fenestração/ramo foram fixadas com stents periféricos cobertos, expansíveis por balão e autoexpansíveis em oito dos 10 ramos-alvo; os dois restantes foram preservados com aberturas e *free flow*. A angiografia pós-procedimento demonstrou o posicionamento correto das endopróteses e dos stents nas artérias viscerais, com permeabilidade mantida, em nove dos 10 ramos-alvo. Entre as complicações, um cateterismo seletivo do tronco celíaco não foi possível na primeira tentativa. Contudo, não houve isquemia mesentérica e, no segundo procedimento, um stent periférico foi implantado com sucesso no tronco celíaco. Os pacientes se recuperaram sem complicações. A TC de acompanhamento demonstrou exclusão completa do aneurisma, sem vazamentos, e perfusão visceral mantida em sete dos oito ramos-alvo. Concluiu-se que o tratamento endovascular de AATA com endopróteses personalizadas é viável e pode ser uma alternativa para pacientes com lesões anatômicas desafiadoras.

## INTRODUCTION

Open surgical treatment of thoracoabdominal aortic aneurysms (TAAA) is associated with high rates of mortality and morbidity.^1,2^ The most common complications of this procedure involve the extensive access to the thoracoabdominal aorta, reimplantation of the reno-visceral vessels, and aortic clamping, which are significantly increased in patients with associated comorbidities, considered to be at high surgical risk.^3^

Since its development, endovascular treatment for TAAA has proven to be a less invasive alternative option with reduced mortality and morbidity rates compared to open repair.^4-6^ In many countries around the world, the number of endovascular procedures performed to treat aortic diseases already exceeds the number of conventional open surgical procedures. In Brazil, the demand for endovascular treatment has grown; however, there are few reports on the medium- and long-term results of these procedures.^6^

Sweet et al.^7^ were the first to present a custom-made endograft with multiple branches that would be anatomically applicable to 88% of patients with TAAA. By the end of 2012, the first commercially available off-the-shelf thoracoabdominal endograft with multiple branches was introduced in Europe.^3^ However, despite these advancements, a significant number of patients with TAAA remain anatomically unsuitable for endovascular treatment.^8^

Endovascular treatment for TAAA requires proximal and distal anchoring zones, but the absence of a suitable proximal and/or distal aortic^9^ neck for endograft placement is the main reason for excluding these patients, as a good aortic neck is necessary for stable fixation to avoid obstructing the major branched vessels. This issue becomes even more significant in patients with aneurysm rupture, in whom the anatomy is typically less favorable for endovascular treatment.^10,11^ Despite this, use of endovascular therapy for TAAA has been expanded over the past decade, even in cases of more complex anatomy, as the procedure is less traumatic. However, studies have raised legitimate concerns regarding the application of endovascular techniques in patients who fall outside the morphological criteria indicated for use of commercial endografts, because mid-term results show low efficacy and high complication rates.^8,10^

The literature includes studies involving modification of commercial endoprostheses within the surgical setting as an alternative strategy for preserving branches in endovascular treatment of TAAA.^12,13^ In this approach, fenestrations are added to commercial endografts to better accommodate and preserve branched vessels, based on each patient’s anatomical characteristics. To design and construct these endografts, computed tomography (CT) imaging data are used to determine the locations for fenestrations based on the visualized vessels. However, several obstacles have been identified regarding widespread use of physician-modified endografts: (a) the preoperative planning time required to measure fenestration locations using CT data; (b) operating room time needed to map the locations of the endograft openings; (c) accuracy of opening placement; and (d) the need to optimize the positioning of all openings to avoid stent struts.^4,11^

In this context, endoprostheses whose designs and construction are adapted according to the specific anatomical characteristics of each patient, when developed through direct interaction with companies in the sector, have yielded good results in the fully endovascular treatment of TAAA.^14^ However, such custom-made endografts are more costly and require extended planning and fabrication periods. Additionally, they are not used for treating patients with acute conditions at risk of rupture.^10^ This endovascular technology for TAAA with endoprostheses is feasible and significantly improves outcomes, justifying indication of the technique for certain specific patient groups selected by surgeons.^14^

The aim of this study was to describe the institution’s first experience with endovascular treatment of TAAA using custom-made endografts, designed and manufactured after obtaining the respective preoperative imaging analyses. This approach shows promise and use of this technique may prove crucial for treating complex cases of patients who are not receiving proper individualized treatment. This study was approved by the Research Ethics Committee at the Faculdade de Medicina de São José do Rio Preto (FAMERP) (CAAE: 84145524.0.0000.5415, Substantiated Opinion: 7.220.650).

## CASE 1

### Part I: Clinical situation

**Figure 1:**
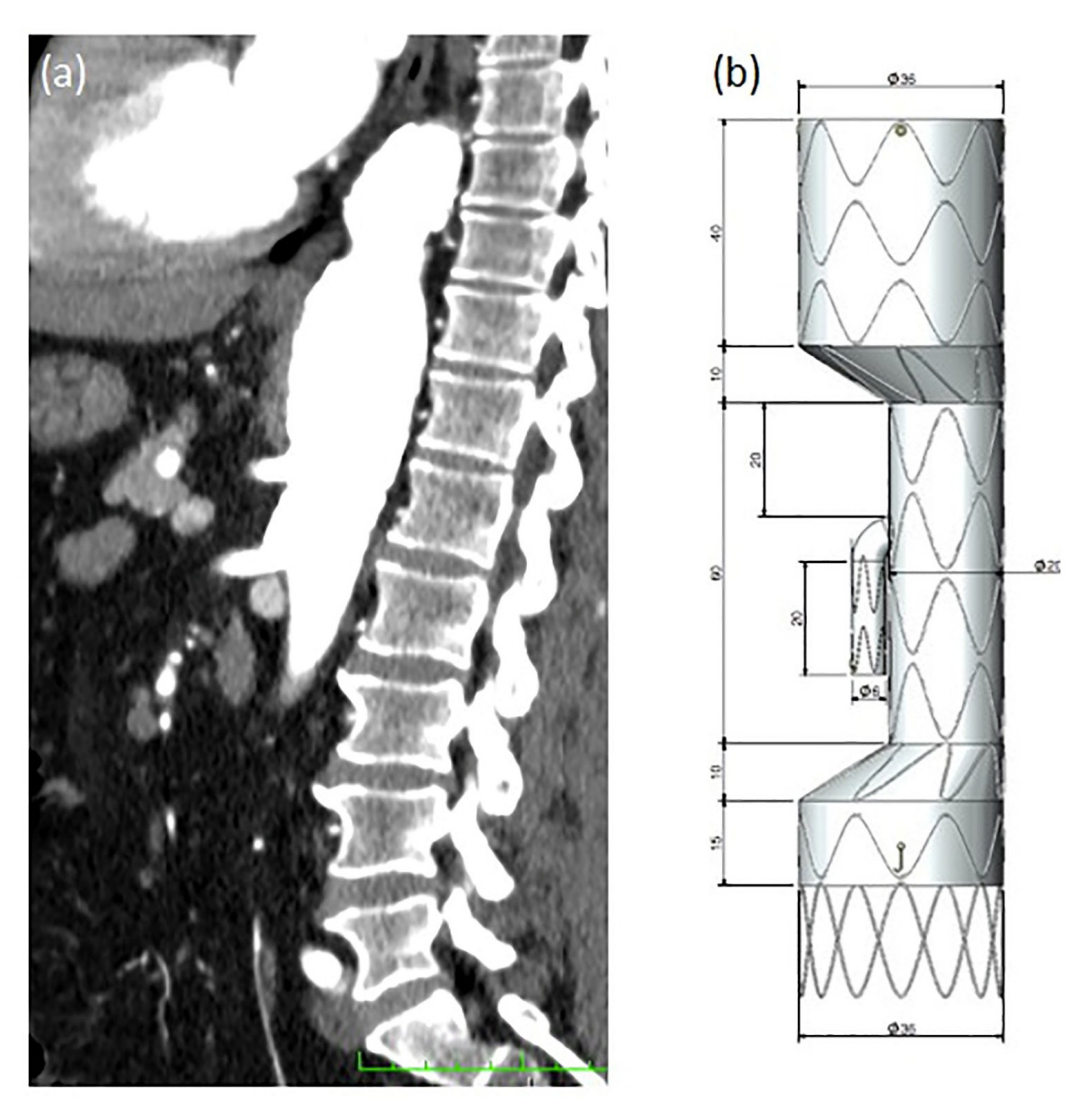
(a) Preoperative computed tomography in coronal view; (b) Design of the custom-made endograft with diameter (36 mm), celiac and mesenteric artery neck (15 mm), celiac trunk branch (8 mm), and distal free flow.

A 44-year-old male hypertensive smoker, was diagnosed with asymptomatic TAAA four years prior to surgery after undergoing a routine examination. The latest imaging analysis indicated aneurysm growth of 0.8 cm, reaching a diameter of 5.8 cm and involving the celiac trunk (Figure 1a). The imaging analysis suggested it would be difficult to fix the endograft at the distal position. For this reason, the free flow technique was used to preserve the superior mesenteric artery and create a branch for the celiac trunk. The endograft used for the treatment was designed with the following characteristics: proximal and distal diameter (36 mm), celiac trunk and mesenteric neck (15 mm), celiac trunk branch (8 mm), and distal free flow (Figure 1b).

### Part II: What was done

**Figure 2:**
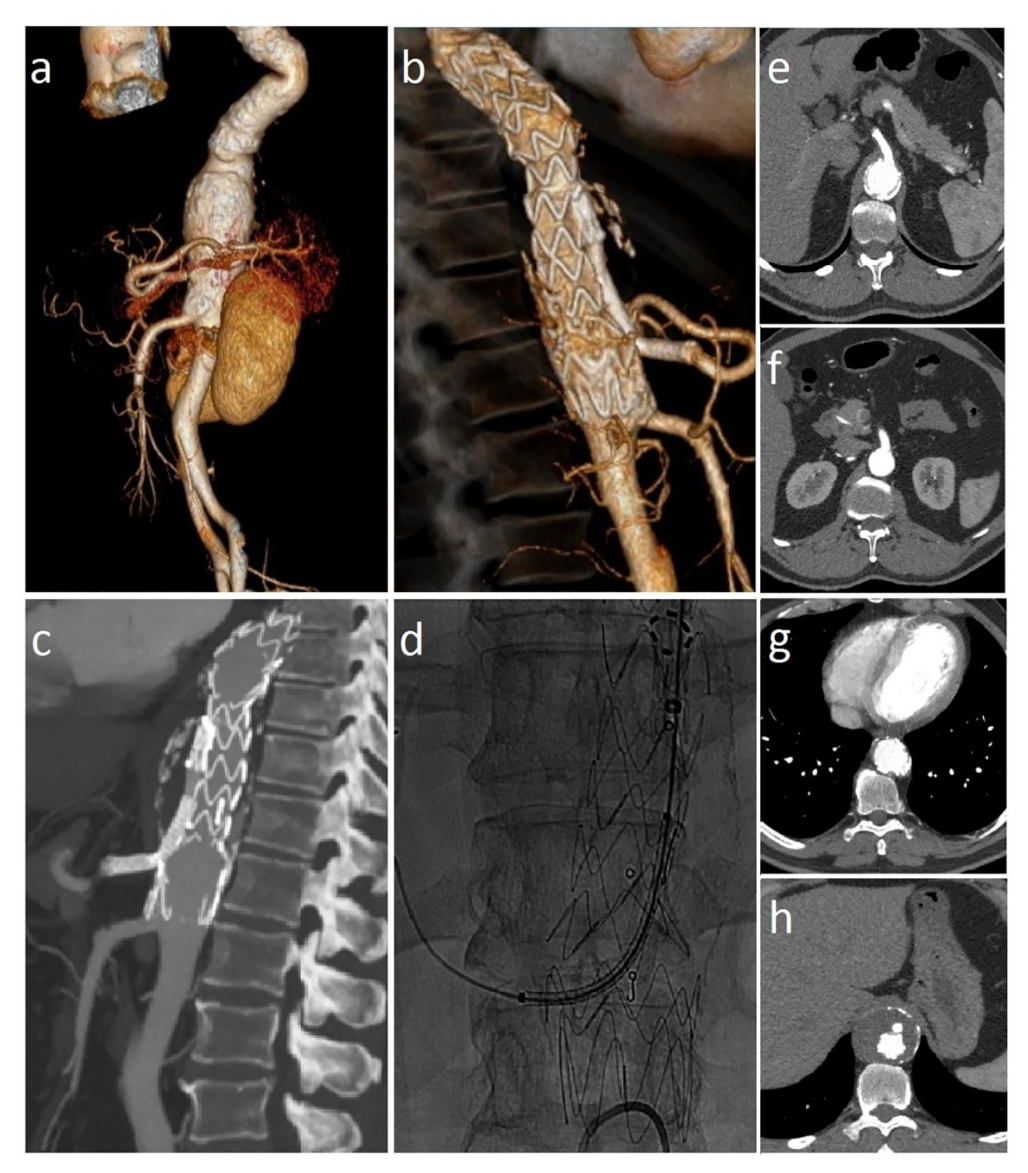
Abdominal aorta angiography of the treated segment in three-dimensional mode. (a) Pre-implantation; (b) Post-implantation (60 days); (c) Correct positioning of the peripheral stent in the endoprosthesis branch and celiac trunk; (d) Post-implantation; well-positioned stents and absence of leaks; (e) and (f) Axial section showing preserved celiac trunk and mesenteric artery; (g) and (h) Axial section showing the absence of leaks.

The pre-procedural angiotomography is shown in Figure 2a. Images acquired 2 months after the procedure show complete exclusion of the aneurysm, absence of leakage, and maintenance of visceral patency (Figures 2b and 2c). The treatment was performed through access via the right femoral artery, with deployment of the main body of the endograft at a position 20 mm above the celiac trunk ostium. Through the left brachial access, the endograft branch was catheterized, followed by placement of the covered Viabahn stent 6 x 100 mm, and then the V12 stent 9 x 59 mm in the endograft branch extending to the celiac trunk (Figure 2d). Angioplasty of the endograft branch and celiac trunk was performed with a Mustang balloon 7 x 40 mm, with contrast infusion for imaging. The total procedure time was 4 hours and 30 minutes.

As a result, angiographic control showed adequate positioning of the main body of the endograft and preservation of the branches of the celiac trunk and superior mesenteric artery (Figures 2e and 2f, respectively), with no leakage (Figures 2g and 2h, respectively).

## CASE 2

### Part I: Clinical situation

**Figure 3:**
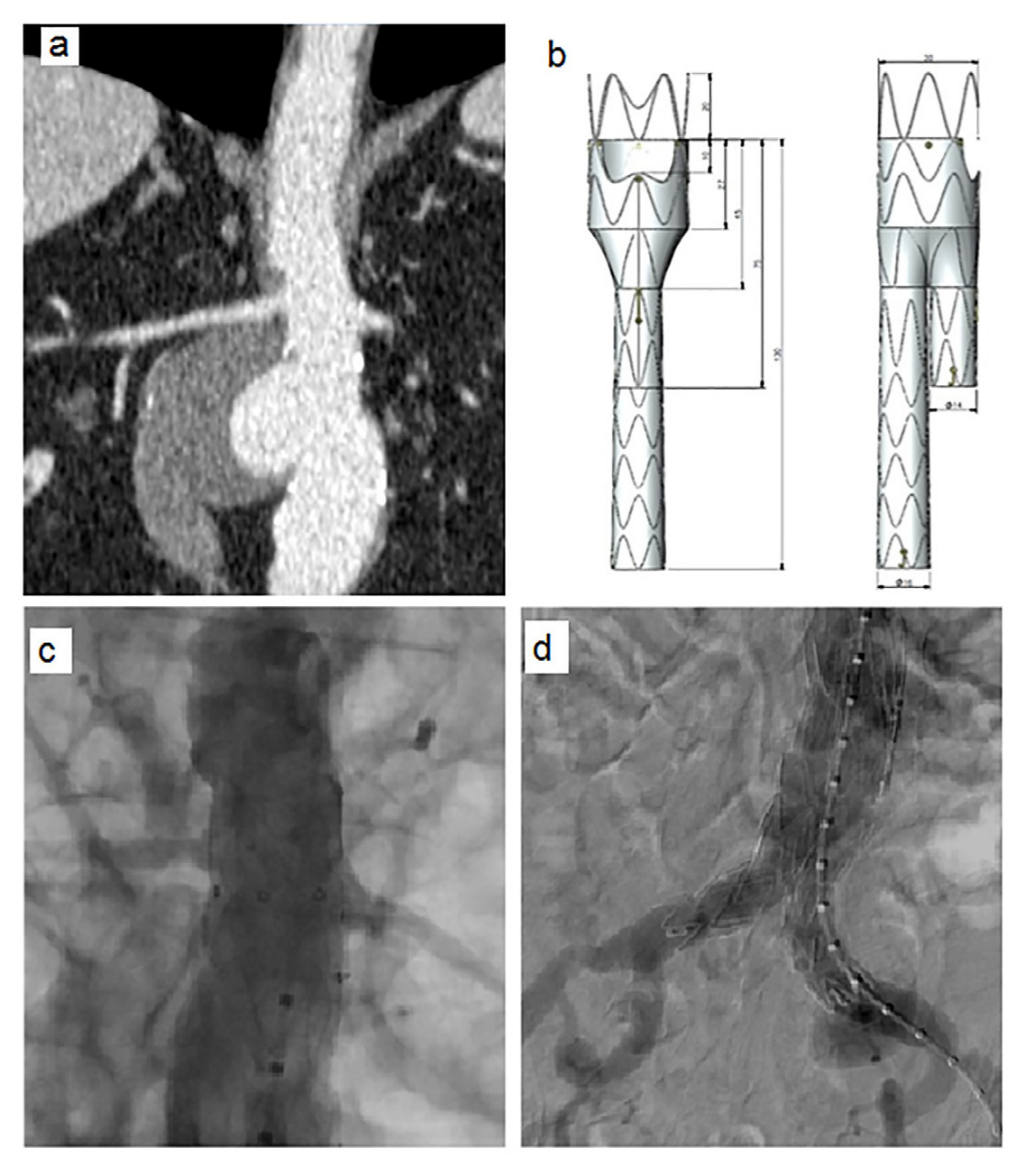
(a) Preoperative computed tomography, coronal view; (b) Illustrative diagram of the custom-made endografts with bifurcated extension (diameter of 30 mm) and free flow / left renal fenestration. After treatment, the thoracic aortic angiography shows the correct positioning of the endoprosthesis; (c) Proximal; (d) Distal.

A 75-year-old hypertensive male with hypothyroidism, emphysema, heart disease, and a pacemaker, presented with abdominal pain. He underwent a CT scan that showed a saccular abdominal aortic aneurysm (Figure 3a). The images revealed a short neck of 7 mm below the left renal artery, which limits the use of a conventional endograft.

Given the complex anatomy and the presence of comorbidities that required meticulous planning to avoid complications – such as the constraints imposed by the pacemaker and the patient’s potential cardiovascular fragility – a fenestrated endograft was selected as the treatment approach. This endograft was specifically designed with a fenestrated window for the left renal artery to ensure adequate renal perfusion and preserve renal function.

### Part II: What was done

The endograft’s specifications were adjusted as follows: a proximal diameter of 30 mm, distal diameters of 15 mm and 14 mm, respectively, and a free flow fenestration for the left renal artery (Figure 3b). This design allowed unobstructed blood flow to the left renal artery, while the right renal neck, measuring 15 mm, provided adequate proximal fixation of the endograft in the desired position.

Use of this fenestrated approach was crucial to achieve secure fixation of the endograft and prevent renal or circulatory complications, particularly given the patient’s challenging clinical condition. The total procedure time was 2 hours and 30 minutes.

Angiographic control showed perfect positioning of the main body of the endograft with maintenance of continuous perfusion to the renal arteries at the proximal (Figure 3c) and distal (Figure 3d) positions.

## CASE 3

### Part I: Clinical situation

**Figure 4:**
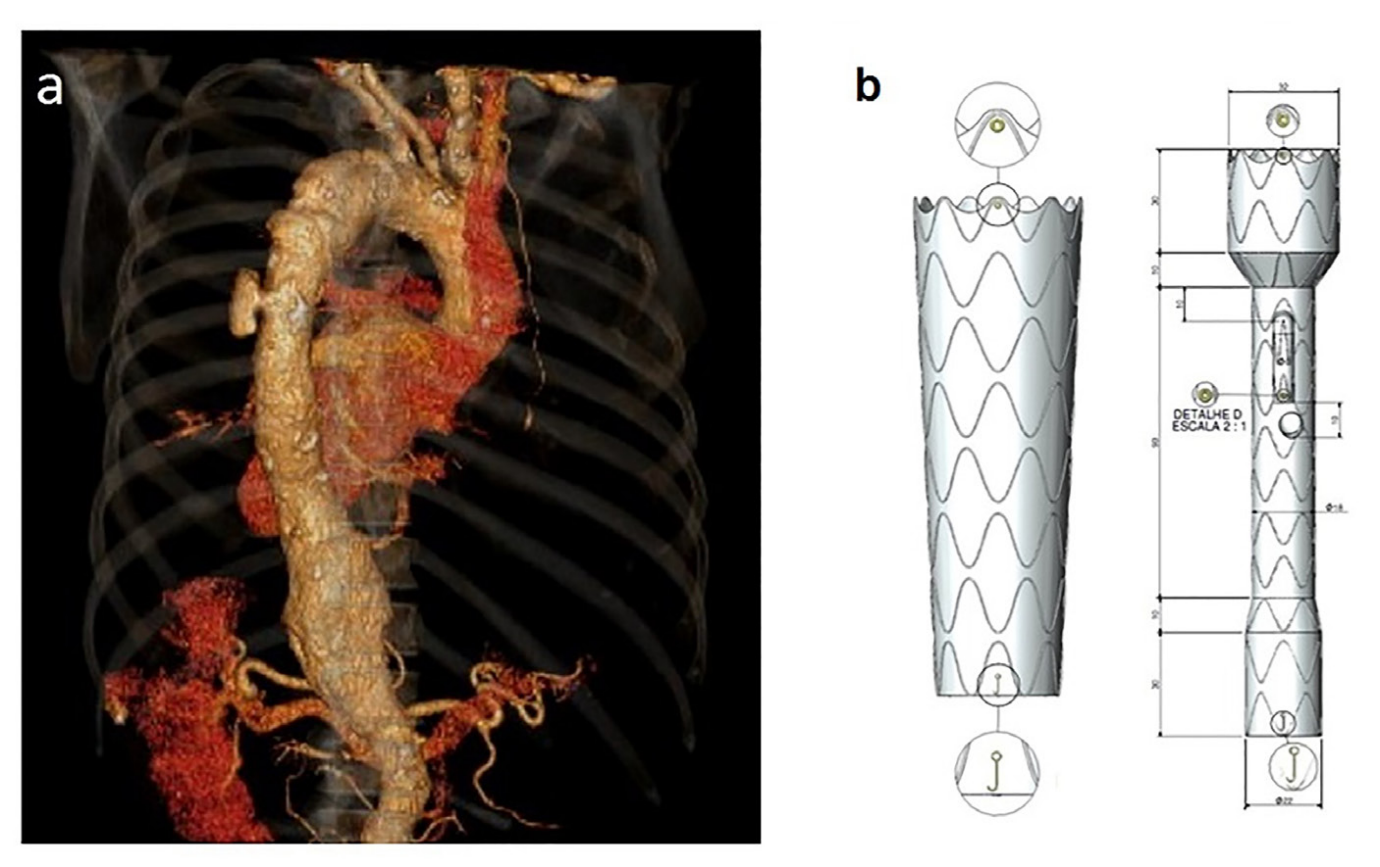
Aortic angiotomography. (a) Ruptured thoracic aortic ulcer; (b) Design of the custom-made proximal conical stent with two branches and renal exclusion.

A 70-year-old male hypertensive chronic renal dialysis patient, presented with chest pain and underwent a CT scan that revealed a ruptured thoracic aortic ulcer. Initially, he was treated as an emergency with a Braile thoracic endograft. Additionally, TAAA was also observed, involving the celiac trunk, mesenteric artery, and extending to the renal arteries (Figure 4a). Two endografts were required to treat this patient: the first designed with a tapered proximal shape with aortic diameters at the proximal (44 mm) and distal (32 mm) implantation sites; the second manufactured with distinct proximal (32 mm) and distal (22 mm) diameters, and custom-made with two branches to preserve the celiac trunk and mesenteric arteries, as the renal arteries were excluded due to the patient being on dialysis (Figure 4b).

### Part II: What was done

**Figure 5:**
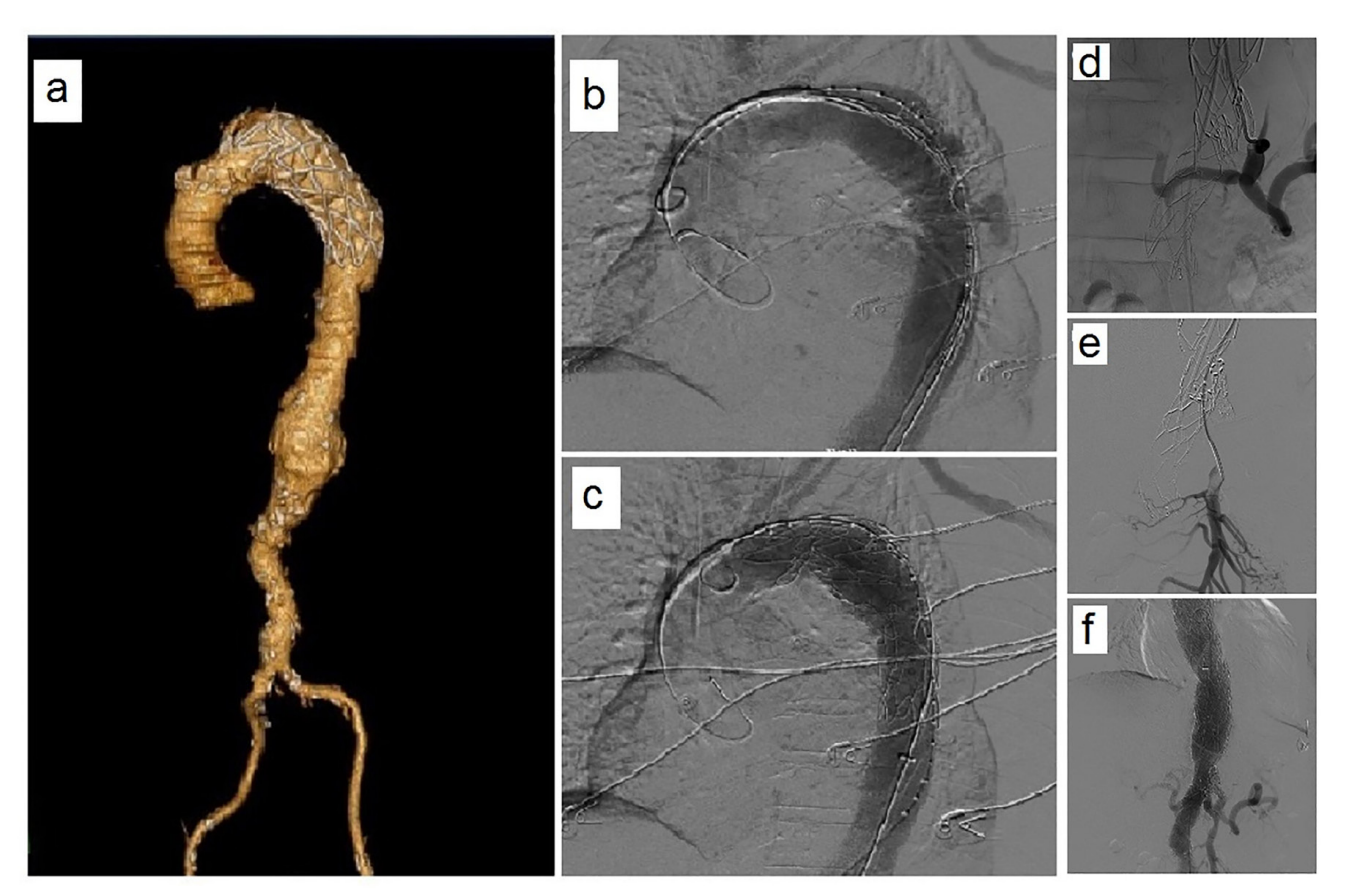
Aortic angiotomography after endoprosthesis implantation and selective catheterization of the visceral branches. (a) Pre-implantation positioning; (b) Post-implantation control of thoracic aorta; (c) Correct post-implantation positioning of the stent graft; (d) Celiac trunk; (e) Superior mesenteric artery; (f) Post-implantation control showing absence of endoleak.

The control angiographic study showed perfect positioning of the main body of the first pre-implant endograft (Figure 5a) and deployment of the main body post-implant (Figure 5b). This control study, after deployment of the endograft, also showed proper positioning of the stent and exclusion of the aneurysm (Figure 5c). Control angiographic analyses showed complete selective catheterization of the visceral branches of the celiac trunk (Figure 5d) and superior mesenteric artery (Figure 5e). Viabahn coated stents measuring 7 x 50 mm and 8 x 50 mm were implanted, followed by a Cook 7 x 40 mm stent in the celiac trunk and superior mesenteric artery, respectively. Additionally, there was no evidence of endoleaks, and the aorta was straight without significant curvature (Figure 5f). The total procedure time was 3 hours.

The patient was discharged from the hospital without any complications. However, he was lost to follow-up at the Vascular Surgery outpatient clinic and phone contact revealed that he had passed away 7 months after the procedure, with the family unable to provide the cause of death.

## CASE 4

### Part I: Clinical situation

**Figure 6:**
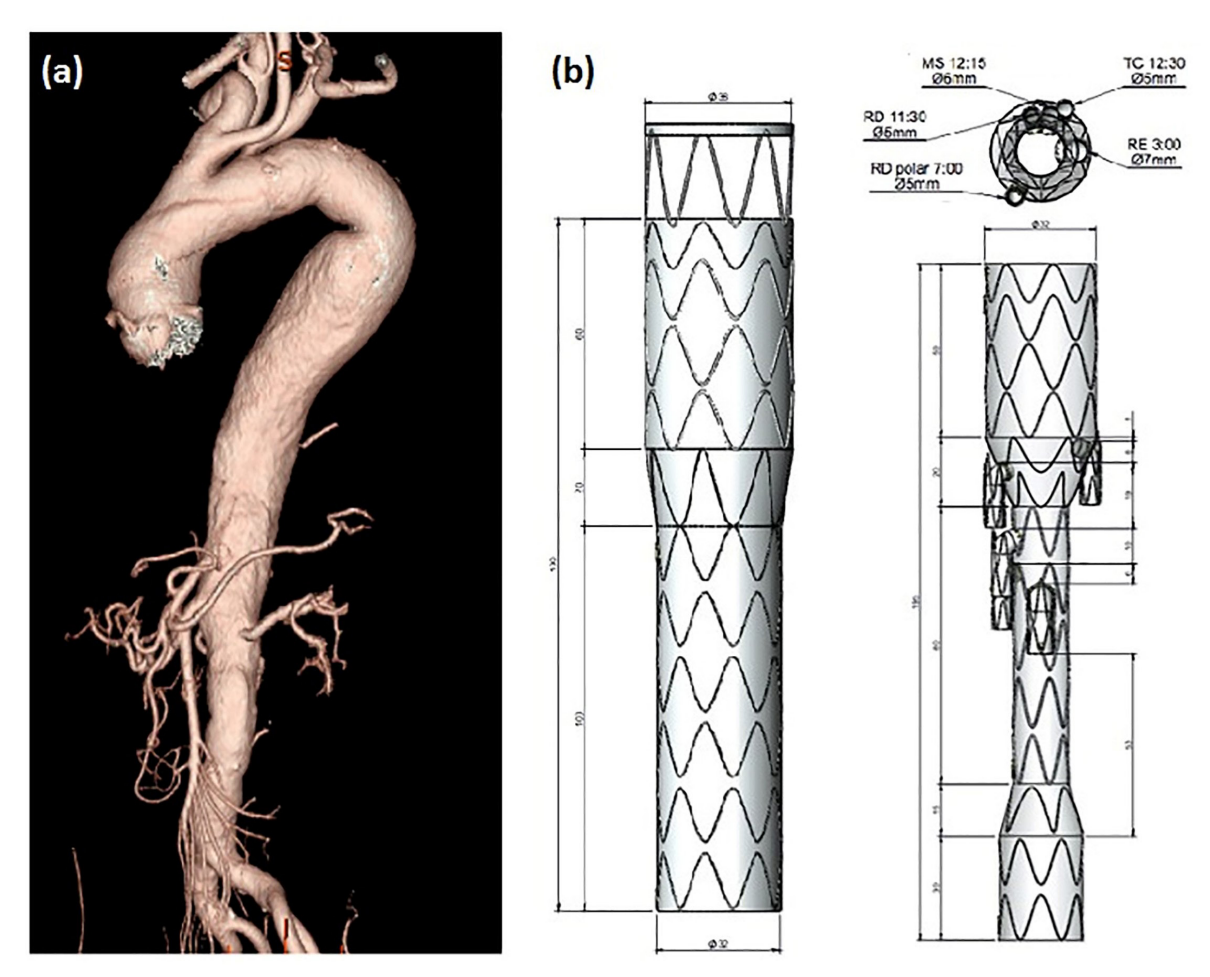
(a) Abdominal aorta angiography in three-dimensional mode of the segment to be treated; (b) Design of the custom-made proximal branched endoprosthesis with five branches.

A 60-year-old female hypertensive smoker. Imaging studies revealed a tortuous aorta with aneurysmal dilation starting in the descending aorta, extending to the infra-renal segment, and involving the visceral arteries: celiac trunk, right renal artery, superior mesenteric artery, and left renal artery (Figure 6a).

### Part II: What was done

Two endografts were used to treat this patient, designed and manufactured with the following characteristics: (1) an endograft with a proximal diameter of 38 mm and distal diameter of 32 mm; (2) an endograft with five branches: right renal artery and right superior polar renal artery, both with diameters of 5 mm, left renal artery, superior mesenteric artery, and celiac trunk (Figure 6b). The first endograft, 38x32/180 mm, was implanted and deployed after the left subclavian artery, and the second endograft, 38x16x24/195 mm, was implanted 2 cm above the respective visceral arteries, with proximal coupling to the previously implanted thoracic endograft.

**Figure 7:**
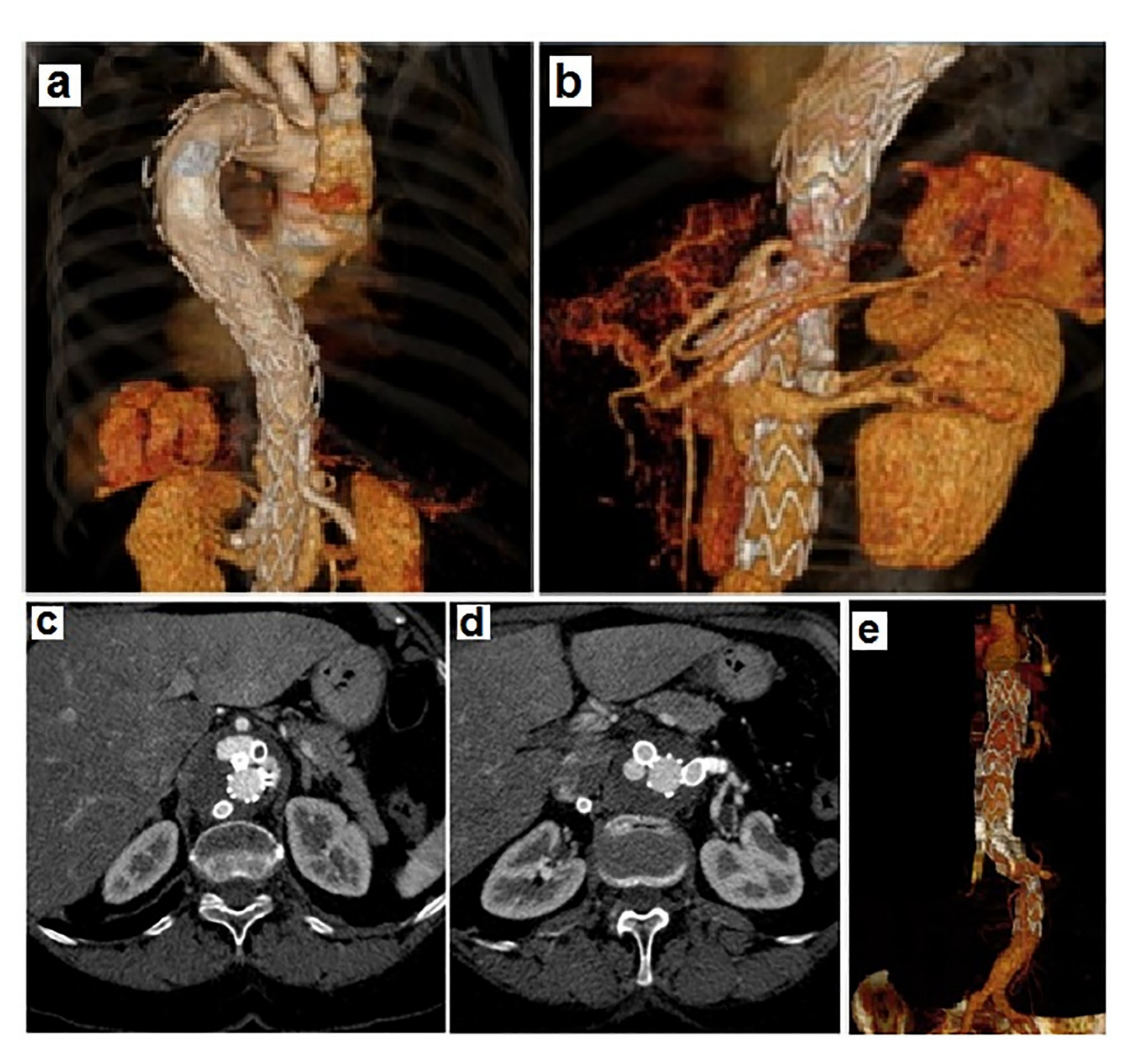
Abdominal aorta angiography, in axial cut. (a) Shows superior mesenteric artery occlusion and (b) Endoleak in the branch of the celiac trunk. Segment of the aorta after surgery, demonstrating good coupling between the stent graft and the peripheral stents; (c) Right and left renal arteries; (d) Endoleak in the celiac trunk; (e) Absence of endoleak in the celiac trunk.

The visceral branches were catheterized through the left axillary access, and covered stents were deployed. Viabahn stents (9 x 50 mm; 10 x 50 mm; 7 x 100 mm, respectively) were implanted in the left renal artery, right renal artery, and right polar renal artery. An Advanta V12 stent was implanted in the superior mesenteric artery. However, it was not possible to catheterize the celiac trunk (Figure 7b). Due to the prolonged surgical time, the procedure was suspended, and the patient was sent to the ICU.

Figure 7 also shows the angiotomography before, after the first surgery, and at the 2-month follow-up after the procedure. Complete exclusion of the aneurysm, absence of leakage, and maintenance of visceral perfusion are evident. After the follow-up CT scan, it was observed that the proximal graft was well positioned. However, as shown in Figure 7c, there was an occlusion of the superior mesenteric artery (likely due to its tortuosity), but no mesenteric ischemia occurred due to the internal leakage in the celiac trunk (Figure 7d). In the second surgery, the internal leakage was effectively treated with placement of covered stents, Advanta V12 8 x 59 mm and Viabahn 7 x 100 mm, in the celiac trunk (Figure 7e).

## DISCUSSION

The use of new fenestration techniques in endografts has enabled endovascular treatment of the entire aorta, including more challenging areas such as the branched vessels of the visceral arteries and the aortic arch. However, there are often some limitations to use of the technique due to significant anatomical variations in these areas, which are difficult to access for endovascular treatment of TAAA.^15^

Studies have demonstrated that Brazilian custom-made endografts are effective for treating TAAA with complex anatomies, achieving high procedural success rates and minimal complications.^4,16^ The custom-made endografts used in the cases presented were manufactured by Braile (São José do Rio Preto, SP, Brazil), according to the individual anatomy of the patients as determined by preoperative CT. The procedures were performed under general anesthesia. Arterial access was obtained by dissection of the common femoral and axillary arteries, both right and left, as required.

Rizza et al.^4^ treated 12 patients between 2022 and 2024, achieving a technical success rate of 91.7%. Only one intraoperative complication occurred, a type III endoleak. All patients were discharged in good health. Follow-up CT scans at 1-3 months showed resolution of an intraoperative leak and two late complications: a type III endoleak and right renal artery occlusion.

Another Italian study used Braile custom-made endografts to treat four patients with complex anatomies, including abdominal and thoracoabdominal aortic aneurysms. Anatomical challenges included an aortic arch angle of 78°, a descending aorta angle of 30°, a 40 mm aortic diameter at the renal level, a 90° alpha angle, a 60° abdominal aorta angle, and a 6 mm iliac access diameter. The study achieved 100% clinical and technical success within 30 days. The average procedure duration was 228 minutes, with no neurological events or renal complications. Postoperative follow-up at 1, 3, 6, and 12 months revealed one case of postoperative iliac artery laceration, treated with a covered stent. After a median follow-up of 16 months, patients were doing well, and CT angiography confirmed aneurysm exclusion and patency of the supra-aortic and abdominal visceral arteries. The authors suggest longer follow-up to validate these promising results.^16^

In clinical practice, this group of patients with anatomic challenges has driven development of custom-made endografts for individualized treatment. This article reports four cases that underwent endovascular treatment with custom-made endografts, two of which (cases 3 and 4) were considered high-risk for conventional surgical repair.

Patient selection and careful preoperative planning, with the participation of the technical team from the endograft manufacturing company, were crucial for the success of the approach. This is because treatment of extensive aneurysms involving the thoracic and abdominal aorta with involvement of the visceral and renal arteries significantly increases complexity and has been a major challenge for surgeons to overcome.

The use of custom-made endografts, as described in this article, offers significant advantages over conventional open repair and also over hybrid open/closed techniques, as it avoids laparotomy or thoracotomy, aortic clamping, cardiovascular instability, and operative blood loss, in addition to reducing the number of visceral ischemia events.^6,17^

One potential disadvantage of the technique is related to the type of arterial access (inguinal and axillary regions), performed through incisions far from the target aneurysm. In this way, certain parameters may limit the perfect execution of the technique in specific cases, such as: excessive tortuosity of the iliac arteries, which limits the manipulation of the catheter release and rotation systems; presence of thick and ostial thrombi; ostial stenosis of the visceral arteries; and, depending on the size of the aneurysm, the space between the stent and the area where the visceral artery emerges may make it difficult to insert secondary bridges and their extensions.^18^

The first reported cases of total endovascular repair of TAAA, by Chuter et al.,^19,20^ were complicated by the patient’s degree of risk and the lack of clinical experience. However, in our study, it was observed that with advances in endovascular techniques and creation of custom-made endografts, there was no evidence of severe complications in the postoperative period in any of the cases presented.

Three of the patients who underwent endovascular treatment for TAAA used branched stents and catheterization of the visceral vessels was necessary to ensure precise alignment of the stent and prevent occlusion of the visceral branch. However, due to the high complexity of the 4th case, some complications occurred, such as occlusion of the superior mesenteric artery, without mesenteric ischemia due to an endoleak in the celiac trunk, which prevented catheterization. The patient’s flow was restored during the second surgery, and, ultimately, the patient remained clinically stable.

Stent migration with subsequent occlusion of the branch has also proved to be an issue in endovascular treatment of the thoracic aorta.^21^ As recommended, in cases 3 and 4, which involved patients with extensive TAAA, a second stent overlap was performed to minimize this migration. Additionally, the diameter of the fixation neck was increased by 15-20%, which provided greater security for fixation at the fenestration/orifice interface.^17^ Another important aspect is that if further endovascular intervention is necessary, the enlargement facilitates access to the vessel.

These are the first institutional reports of treatment for TAAA performed entirely percutaneously, preserving the visceral branches through the use of custom-made endografts tailored to the specific anatomical characteristics of each patient. The high quality of preoperative imaging to enable stent customization, the expertise of the stent development team, and clinical experience were essential for the success in treating these complex aneurysms. The CT images acquired during the 2 months of follow-up of the three surviving patients demonstrated complete exclusion of the aneurysm and patency of the main stent and its branches (except for the occluded superior mesenteric artery branch in the patient described in case 4). The effectiveness and durability of the technique will require validation in a clinical series with a larger number of cases.

Technological advances in the development of standard stents are not sufficient to treat all available scenarios, especially in specific patients. This study showed good results using Braile custom-made stents for endovascular treatment of TAAA and offers a viable and promising alternative option for treating patients with lesions in challenging anatomies.

## Data Availability

All data generated or analyzed are included in this article and/or in the supplemental material.
